# Fault Identification in Membrane Structures Using the Hilbert Transforms

**DOI:** 10.3390/s22166224

**Published:** 2022-08-19

**Authors:** Aleksandra Waszczuk-Młyńska, Adam Gałęzia, Radkowski Stanisław

**Affiliations:** Faculty of Automotive and Construction Machinery Engineering, Warsaw University of Technology, Narbutta 84 St., 02-524 Warsaw, Poland

**Keywords:** Hilbert transform, generalized Hilbert transform, spectral moments, damage detection, membrane structure

## Abstract

Fault diagnostics present a crucial technical issue in the areas of both the condition monitoring of machines and the monitoring of structural health. The identification of faults at an early stage in their development has an immense effect on the safety of monitored structures. Correct identification allows for the monitoring of the development of faults and the choosing of optimal operation strategies. This article discusses a method of monitoring structural health, based on the application of the Hilbert transforms (GHT and FrHT) for detecting fault formations and their development in membrane structures. A signal resulting from the HT is analyzed using spectral analysis to identify features indicating the technical state.

## 1. Introduction

The current development of structural health monitoring (SHM) tools in the domain of technical diagnostics is directed towards creating methods enabling the detection of damage at a possibly early stage, among other aims. At present, the emergence of a defect does not exclude a structure from use. If a failure is detected, identified, and located early, and if its development is under constant monitoring, the structure can continue to be operated at a specified level of safety.

According to the present state of technology, the origination of a defect does not preclude further use of a structure. However, further operation of an structure, in accordance with a given level of safety, implies the need for the early detection, identification, and localization of the fault. This tactic should be combined with the control of the fault’s development and the performance of suitable repair actions. The detection of an early phase of a fault allows us to change operational conditions or to plan maintenance and repairs [1,2].

The chief merit of the present research and development work is the analysis of the possibility of using a transition transform operation based on planned maintenance actions towards a strategy of operation based on the structure’s technical state [3]. The identification of damage using predictive models allows the practical implementation of a proactive strategy for the operation of machines and structures by implementing conscious changes in the operational conditions and planned maintenance [3]. Many different approaches focus on the identification of the early stages of faults, among which, the following can be included: modern techniques of signal processing [4,5,6,7,8,9]; unconventional measurement solutions, such as using sensors embedded in the monitored structure [10]; the use of magnetic phenomena to detect symptoms of damage [11,12,13,14,15]; and the application of acoustic emissions [16].

The main requirements for the developed methods dedicated to the detection of early phases of damage are reliability, resistance to disturbances in measurements, low cost (in particular, costs arising from computation), and ease of result interpretation. Often, these requirements entail the examination of various approaches and analyses to determine the scope of the applicability of the methods. Signals recorded from the machine or structure undergo processing to extract features that indicate the object’s technical state [3]. Taking that into consideration leads to an interesting issue of the detection of the early stages of damage development within structures, such as those using plate and shell technology. The widely discussed and presented detection methods, referring to Lamb wave analysis, allow us to obtain satisfactory results while meeting previously adopted assumptions.

First of all, there is an assumption that has been made regarding the possibility of using many sensors directly installed on the tested object, as well as the assumption that there exists a possibility of stimulating vibrations, most often high-frequency, with the use of piezoelectric sensors in conjunction with the simultaneous use of advanced methods of signal analysis [17,18].

In many cases, these conditions are difficult to meet, especially in the study of membrane-type structures characterized by low bending-stiffness, which means that the mounting of piezoelectric sensors may cause significant disturbances in the generated dynamic response of the tested system.

Similar disturbances are introduced by a set of sensors measuring the structure of waves reflected off of an obstacle, i.e., a developing fracture. In order to meet these difficulties, while possibly reducing costs, this paper deals with the problem of crack-detection based on the measurement and analysis of low-energy disturbances in the vibration signal as measured and recorded without contact at a distance using a laser Doppler meter. Taking into account the vibration patterns of the object, in this case the diaphragm, a decision was made to conduct measurements at the point where the observation of the first and second modes of vibration is possible. The vibrations were caused by acoustic stimulation from a loudspeaker in the frequency range of 20–400 Hz. The selected feature, or set of features, must be diagnostically informative and immune to disturbances occurring during measurements. Features that are optimal in regard to the diagnostic task are often used in autonomous diagnostic systems [3].

One of the widely used methods of signal processing is the Hilbert transform [19], which has been successfully applied to many tasks involving technical diagnostics [20]. Most frequently, the Hilbert transform has been used to determine the instantaneous amplitude disturbances and instantaneous signal-frequency changes. For example, determining the parameters modulating the carrier signal enables the identification of the bearing faults [21].

This article presents a comparative analysis of the methods for signal-analysis based on the generalized Hilbert transform and the fractional Hilbert transform to detect damage in membrane structures. Faults emerging in such systems, particularly at an early stage of their development, manifest themselves through subtle energy changes. Although emerging faults affect the membrane’s rigidity, the changes in natural frequencies are very weak [22]. For this reason, the method of fault-detection which relies solely on changes in the frequencies of natural vibrations is not practically applicable. Nevertheless, the occurrence of modulation phenomena around the membrane’s resonant frequencies may be used in the process of determining the technical state of the analyzed object. Emerging envelope disturbances can be viewed as symptoms of damage. Some of the most common features enabling the determination of changes in the signal spectrum are spectral moments. They are used in technical diagnostics as features characterizing the changes caused by the shift of energy between the frequency bands of the diagnostic signal, which proves the development of damage [11,22,23,24].

The methodology for the determination of the technical state of the membrane presented in this paper is based on the analysis of spectral moments calculated for the spectra of the generalized and fractional envelopes of the vibration signal.

Section 2 presents the object of analysis and the measurement equipment used for the experimental investigations. Section 3 gives a brief theoretical background on the ideas, terminology, and transforms used in the present research. Subsequently, Section 4 presents the application of the developed method for the analysis of the experimental data. Section 5 contains a discussion and the conclusion.

## 2. Presentation of the Hilbert Transforms and Spectral Moments

This section mainly presents theoretical issues regarding the Hilbert transforms, as well as various tools originating from its concepts. The presented tools were used to analyze signals to detect faults in the circular membrane.

### 2.1. Classical Hilbert Transform

The Hilbert transform (HT) [24,25] is an integer transformation mapping a given real signal into a complex signal. A characteristic feature allowing for the differentiation between the Hilbert transform and other transforms, such as the Fourier or Laplace transforms, is that the result of the transformation is located in the same domain as the transformed signal—the function of the variable t is transformed into the other signal (a different function), which is also dependent on *t*.

Equation (1) shows the Hilbert transform of the real signal:

(1) $$y\left(t\right)=H\left\{x\left(t\right)\right\}=\frac{1}{\pi }\int_{-\infty }^{\infty }\frac{x\left(\tau \right)}{t-\tau }d\tau$$

where *x*(*t*) is the transformed real signal.

The analytical signal for the currently considered signal *x*(*t*) is described by this formula:

(2) u(t)=x(t)+iy(t)

The obtained result is a complex signal in which the real part Re *u*(*t*) is a transformed signal *x*(*t*), and the imaginary part Im *u*(*t*) is the Hilbert transform *y*(*t*) of the transformed signal *x*(*t*).

### 2.2. The Generalized Hilbert Transform

The generalized Hilbert transform (GHT) for the signal *x*(*t*) is described by Equation (3) [26]:

(3) $$y_{\alpha }\left(t\right)=H_{\alpha }\left[x\left(t\right)\right]=\frac{e^{-ia\left(\alpha \right)t^{2}}}{\pi }\int_{-\infty }^{\infty }\frac{x\left(\tau \right)e^{ia\left(\alpha \right)\tau^{2}}}{t-\tau }d\tau \;\mathrm{for}\;\;\alpha \neq 0,\frac{\pi }{2},\pi$$

where $a\left(\alpha \right)=\frac{cot\alpha }{2}$.

The generalized analytical signal of the generalized Hilbert transform can be described using Equation (4):

(4) $$u_{\alpha }\left(t\right)=x\left(t\right)+iH_{\alpha }\left[x\left(t\right)\right]$$

Similar to the concept of envelopes and instantaneous frequency, a generalized envelope can be calculated for a given *α*.

### 2.3. The Fractional Hilbert Transform 

The fractional Hilbert transform (FrHT) originates in the work of Gabor [27], but the form shown below (5) results from the works of Venkitaraman and Seelamantula [28]:

(5) $$y_{\varphi }\left(t\right)=H_{\varphi }\left[x\left(t\right)\right]=cos\varphi x\left(t\right)+sin\varphi H\left[x\left(t\right)\right]=cos\varphi x\left(t\right)+\frac{1}{\pi }\int_{-\infty }^{\infty }\frac{sin\varphi }{t-\tau }x\left(\tau \right)d\tau =(h_{\varphi }*x)\left(t\right)$$

where $-\frac{\pi }{2}\leq \varphi \leq \frac{\pi }{2}$, and $h_{\varphi }=cos\;\varphi \delta \left(t\right)+sin\varphi \frac{1}{\pi t}$.

For $\varphi =\frac{\pi }{2}$, the fractional Hilbert transform is equal to the Hilbert transform (1), which ensures that $y\left(t\right)=y_{\varphi }\left(t\right)$.

The analytical signal for FrHT is created as follows (6):

(6) $$u_{\varphi }\left(t\right)=x\left(t\right)+e^{i\left(\pi -\varphi \right)}H_{\varphi }\left[x\left(t\right)\right]$$

After the twofold iteration of the Fourier transform and minor transformations, the following formula can be obtained:

(7) $$u_{\varphi }\left(t\right)=sin\;\varphi e^{i\left(\frac{\pi }{2}-\varphi \right)}u\left(t\right)$$

This equation is the most common form of the FrHT, frequently seen in literature on the subject [29].

### 2.4. Spectral Moments

Spectral moments are parameters defining the shape of the signal spectrum. The *m*-th-order spectral moment is expressed by Equation (8) [11,23,24]:

(8) $$M\left(m\right)=\sum_{f_{i}}\left|G\left(f_{i}\right)\right|{\left(f_{i}\right)}^{m}$$

where $G\left(f_{i}\right)\;$ is the value of the signal spectrum for frequency $f_{i}$.

The zeroth spectral moment is often used to normalize the moments of higher orders and is interpreted as the average amplitude of the spectrum. Most commonly, normalized spectral moments are used. The normalized spectral moment of the *m*-th order is expressed by Equation (9):

(9) $$M_{u}\left(m\right)=\frac{M\left(m\right)}{M\left(0\right)}$$

The first-order normalized moment is interpreted as the center of gravity of the spectrum.

## 3. The Membrane Structures and the Experimental Setup

### 3.1. Membrane Structures

The membrane structures are thin, flexible spatial surfaces. Their wide range of applications, from the roofing industry, to the environmental protection, food, textile, medical, and acoustic industries, cause membrane structures to be an interesting research object. The specific properties of such structures allow their application in energy recuperation [30]. The membrane structures, and in particular the membranes, require suitable diagnostic methods which are able to detect faults in such delicate structures. As an object of investigation, a circular membrane was chosen for this study.

### 3.2. Test Stand

**Figure 1:**
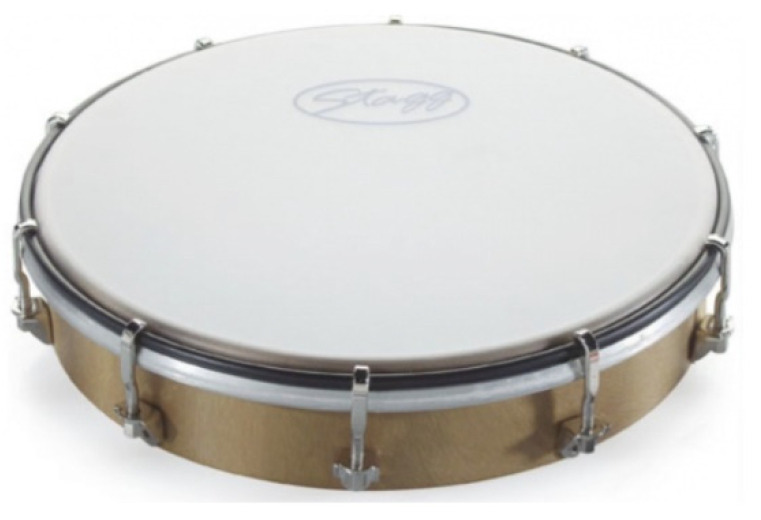
Measurement object.

The plastic, round membrane structure (Figure 1), with a diameter of 10 inches (equal to 254 mm), was mounted flexibly in rectangular frame. The acoustic excitation caused the object to vibrate. The excitation was white noise in the frequency range of 20–500 Hz.

**Figure 2:**
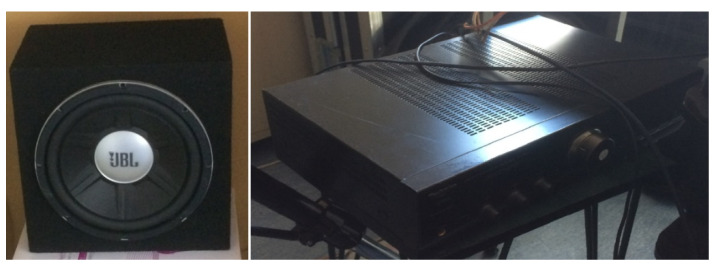
Measurement tools and research stand, the left low-tone JBL GTO 1214 loudspeaker, the right Technics SU-A700 stereo amplifier.

The acoustic signal was generated by a low-tone JBL GTO 1214 loudspeaker, possessing a maximum power of 1400 W and bandwidth of 20–400 Hz, and a Technics SU-A700 stereo amplifier, possessing an output power of 45 W per channel and bandwidth of 20–20,000 Hz, as shown in Figure 2.

**Figure 3:**
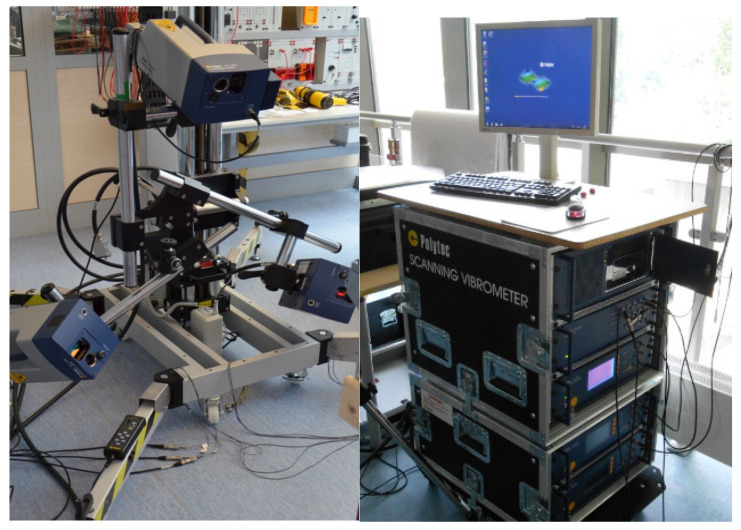
Scanning laser vibrometer.

The vibrational response of the membrane structure was recorded using a scanning laser vibrometer (Figure 3). This device allowed for contactless measurement which, in the case of the considered structure, made it possible to avoid disturbing the structure’s stiffness, which would have been caused by the mounting of the additional mass.

The measurements were recorded for 3 test cases: an undamaged membrane (designated as “UN”), a structure with circular damage measuring 5 mm in diameter and located in the center of the membrane (designated as “U5”), and a structure with circular damage measuring 10 mm in diameter and located in the center of the membrane (designated as “U10”).

## 4. Application of the Transforms and Spectral Moments to the Experimental Data

**Figure 4:**
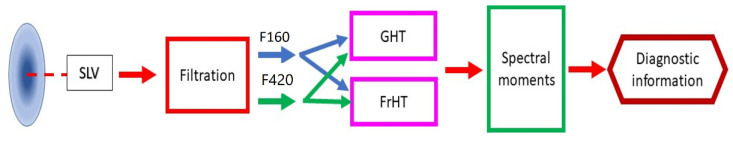
Diagram of the Method.

Below, the experimental results are presented. The investigated object was a circular membrane tested in 3 cases: with no damage (UN), with damage measuring 5 mm in diameter (U5), and with damage measuring 10 mm in diameter (U10) in its central part. For each considered case, the diagnostic information was extracted via filtration of the system’s response around the first and second frequency of natural vibrations. The filters designed for this purpose have been designated as “F160” (first natural frequency) and “F420” (second natural frequency), respectively, in this paper. The extracted signals were analyzed using the respective Hilbert transforms, and their spectral moments were computed. Figure 4 illustrates the workflow of the performed analysis.

### 4.1. Applications of the Fractional Hilbert Transform

**Figure 5:**
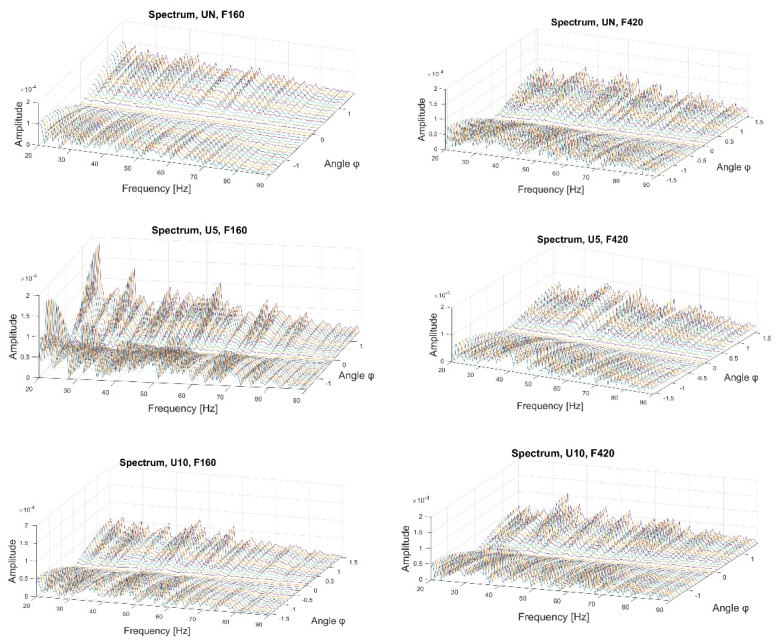
Envelope spectrum of the fractional Hilbert transform.

First, the data after filtration was processed using the fractional Hilbert transform. It allowed us to obtain the envelope spectra of the analytical signal for various angles *φ*. As shown in the subsequent rows of Figure 5, all of the variants of the technical state are presented from top to bottom: UN, U5, and U10. The first column of the plots represents the envelope spectra for the first vibration form, while the second column represents those for the second vibration form. The three-dimensional plots illustrate the relationship between the amplitude, the frequency, and the angle *φ*.

It can be observed that, along the growth of the damage, new characteristic frequencies emerged, a fact which is even more evident with the second form of vibration. The greater the damage, the greater the shift to the right of the additional frequencies, which means that they increase. Moreover, the change in the value relative to the angle *φ* adjusts the plot’s scale.

### 4.2. Application of the Generalized Hilbert Transform

**Figure 6:**
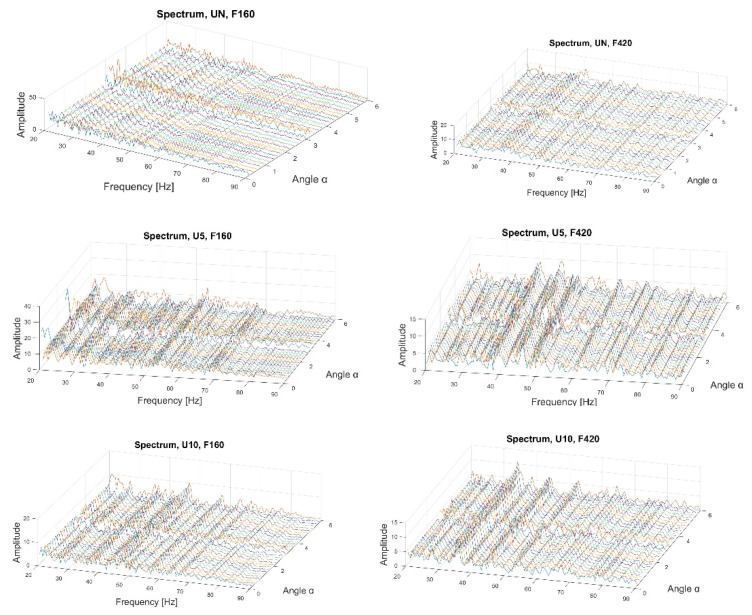
Envelope spectra of the generalized Hilbert transform.

Figure 6 illustrates the envelope spectra of the analytical signal obtained using the generalized Hilbert transform. As in the previous plots, the rows present various technical states, whereas the columns show the results of the filtration around the first and second natural frequencies. The presented envelope spectra depend on the angle *α* and the frequency.

By analyzing the plots in Figure 6, we can observe that different types of damage influence signal bands around the first and second forms of vibration. Similar to the case of the fractional Hilbert transform, new characteristic frequencies emerge, the number of which increases with increasing damage. For the first form of vibration for the undamaged element, the plot for the frequency above 20 Hz is rather smooth, whereas, with the appearance of damage, the plot seems distorted by noise, and furthermore, for the damage with a diameter of 5 mm, an additional frequency of approximately 55 Hz emerges; for the damage with a diameter of 10 mm, additional spectral components of 40 Hz and 50 Hz emerge.

While considering the second form, the growth of the amplitudes of the characteristic frequencies becomes visible, and depending on the damage, a larger number of characteristic frequencies appear.

### 4.3. Application of Spectral Moments with the Fractional Hilbert Transform

**Figure 7:**
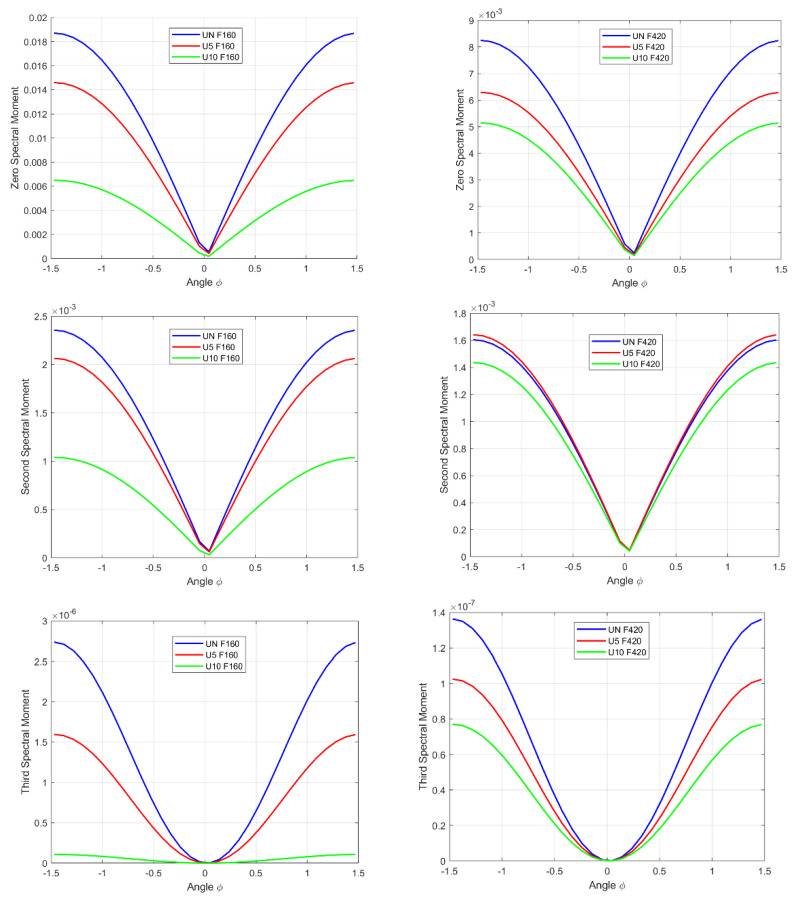
Spectral moments for the fractional Hilbert transform.

In the next step of our analysis, spectral moments for the previously obtained data, i.e., those corresponding to each of the envelope spectra of the analytical signal obtained with the use of the fractional Hilbert transform, were computed as functions of the angle *φ*. Figure 7 shows the zeroth, second, and third spectral moments because the first moment is independent of *φ*.

The first normalized spectral moment of the first vibrational form is equal to:

$$M_{u}\;{\left(1\right)}_{UN}=14.2\;M_{u}\;{\left(1\right)}_{U5}=17.5\;M_{u}\;{\left(1\right)}_{U10}=22$$

The first normalized spectral moment of the second vibrational form equals:

$$M_{u}\;{\left(1\right)}_{UN}=26.9\;M_{u}\;{\left(1\right)}_{U5}=31.8\;M_{u}\;{\left(1\right)}_{U10}=32.5$$

Because the first spectral moment is the spectral center of gravity, its independence of *φ* proves that the fractional Hilbert transform is data-rescaling. Additionally, together with the increasing damage size, the values of the moments grow for the first and second vibration forms.

### 4.4. Application of Spectral Moments with the Generalized Hilbert Transform

**Figure 8:**
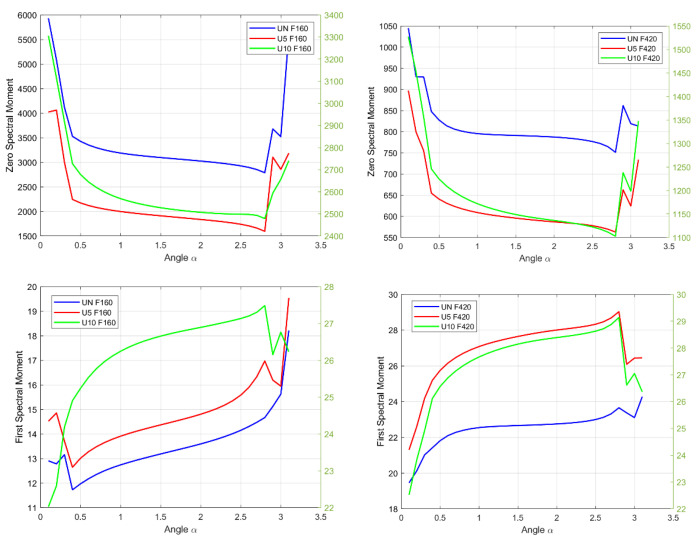

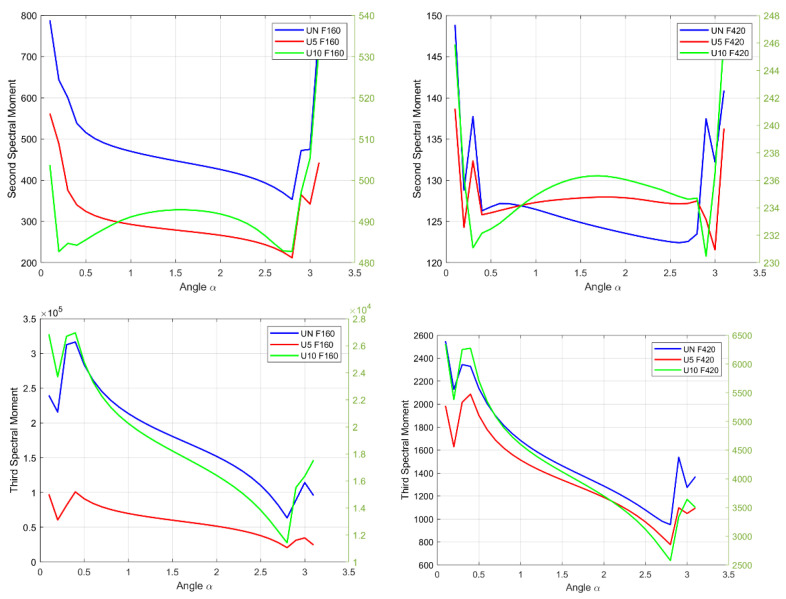
Spectral moments for the generalized Hilbert transform; the left axis indicates UN and U5, and the right axis indicates U10.

In the following section, spectral moments are considered, but they are calculated for the envelope spectrum obtained by the generalized Hilbert transform. Figure 8 shows the successive moments, from the zeroth order to the third order, for the first and second vibrational forms. An analysis of these moments enables the identification of not only quantitative fluctuations, which were evident earlier, but also qualitative changes observed in the case of a damaged object. Above all, the moments that indicate the damage are the first spectral moment at the first vibrational form and the second moment at the second vibrational form.

The first spectral moment for F160, at a value of approximately *α* = 2.765, changes its waveform character; in the undamaged case, the curve of the spectral moment rises; when the damage emerges, the values begin to decrease to approximately *α* = 2.985, and then they start increasing again.

The second spectral moment for F420 also changes the waveform character for the undamaged element. For *α* ∈ (0.503, 2.765), the function plot monotonically decreases, and when the damage occurs, regardless of the size, the function in this range begins to increase. On the other hand, in the interval *α* ∈ (2.765, 3.10), the signal obtained from an undamaged membrane grows first, while that obtained from a damaged membrane drops sharply, only to increase again in the next step. Otherwise, damage shifts the angle at which the trend changes signal an increase.

## 5. Conclusions

The Hilbert transforms of both types, generalized and fractional, can be used in diagnosing faults in elements, such as membranes, because failure causes additional characteristic frequencies to emerge in the envelope spectrum.

The use of the spectral moments of the analytical signal envelope spectrum obtained with the fractional Hilbert transform yields information about the damage when the values of the moments are observed and compared. With the occurrence of damage, the value of the moments increases, as it also increases along with the growing damage size.

Spectral moments, computed on the basis of the analytical signal envelope spectrum of the generalized Hilbert transform, yield qualitative results regarding damage. Because of these moments, the unequivocal determination of whether damage has occurred within the tested object is possible without knowledge of the previous history of the object.

**Figure 9:**
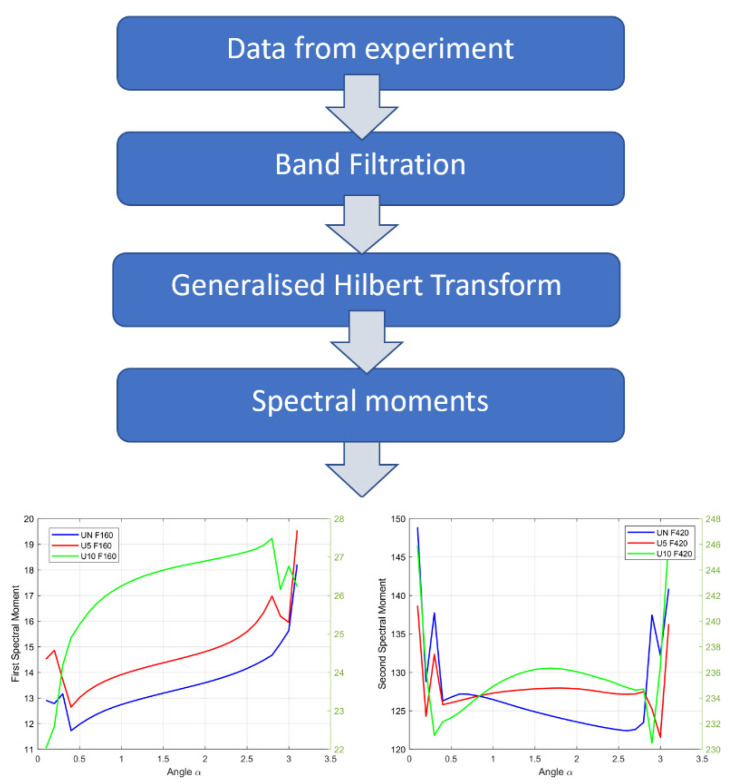
Diagram of the method for fault-detection.

Because the performed research indicates that for low frequencies of interest, discontinuities exist [31], in the presented method, only the first and the second natural frequencies were used. In contrast to other methods, the presented fault-detection method provides qualitative diagnostic information which allows for the evaluation of the technical state (Figure 9).

It should be noted that TH is the basic source of information about the occurring damage and its development in the membrane, which is confirmed by the dependence of the fractional TH spectral moments of the tested signal—the greater the contribution of TH (the greater the absolute value of the *φ* angle in the presented graphs) in the structure of the analyzed signal, the clearer the information about the occurrence of damage is. However, only the spectral moments of the generalized TH, and especially the analysis of the results obtained for the extreme values of the *α* ∈ {0, π} angle interval, allow one to observe a qualitative change in the value of the spectral moments (respectively, the first moment for the first vibration’s eigenfrequency, and the second moment for the second vibration’s eigenfrequency) at the moment of the occurrence of damage. This observation can be the basis for building a qualitative model of the diagnostic inference procedure, as shown in Figure 9.

## Data Availability

Not applicable.
